# Exploratory clinical and urodynamic changes associated with mirabegron-containing multimodal treatment in children with non-monosymptomatic nocturnal enuresis: a retrospective real-world study

**DOI:** 10.3389/fped.2026.1866454

**Published:** 2026-08-05

**Authors:** Zhenxian Cai, Jiangcun Silang, Xue Ma, Dunzhu Gesang

**Affiliations:** 1 Department of Urology, The First Affiliated Hospital of Xizang University (The Second People's Hospital of Xizang Autonomous Region), Lhasa, China; 2 Department of Urology, People’s Hospital of Xizang Autonomous Region, Lhasa, China; 3 Department of Urology, West China Hospital of Sichuan University, Chengdu, China; 4 Department of Neurosurgery, The First Affiliated Hospital of Xizang University (The Second People's Hospital of Xizang Autonomous Region), Lhasa, China

**Keywords:** bladder compliance, children, lower urinary tract symptoms, mirabegron, non-monosymptomatic nocturnal enuresis, overactive bladder, pediatric urology, urodynamics

## Abstract

**Background:**

Evidence regarding mirabegron-containing treatment for children with non-monosymptomatic nocturnal enuresis (NMNE) is limited, and its independent effects require controlled evaluation.

**Objective:**

To describe exploratory clinical and urodynamic changes observed after mirabegron-containing multimodal treatment in children with NMNE.

**Methods:**

We retrospectively reviewed 31 children with NMNE who received mirabegron-containing treatment at the outpatient clinic of West China Hospital of Sichuan University between July 2024 and March 2025. Mirabegron was added to first-line treatment when voiding diaries suggested reduced functional bladder capacity. Patients received 25 or 50 mg once nightly according to age and weight for 12 weeks, without dose adjustment. Voiding diary parameters and available urodynamic measurements were compared before and after treatment.

**Results:**

After 12 weeks, voiding frequency, daytime incontinence episodes, and nocturnal enuresis episodes decreased, while maximum voided volume increased. Maximum cystometric capacity was measured before and after treatment in all 31 patients and showed a modest within-cohort increase. Follow-up measurements of detrusor pressure at the end of filling and bladder compliance were available for only four patients. These descriptive data showed lower detrusor pressure and higher bladder compliance after treatment. Based on nocturnal enuresis episode reduction, 28 of 31 patients (90.3%) met the predefined diary-based improvement threshold. This proportion cannot be interpreted as a pharmacologic response attributable to mirabegron because treatment was multimodal and uncontrolled. No treatment-related adverse events were documented, but retrospective records are insufficient to establish safety.

**Conclusion:**

Mirabegron-containing multimodal treatment was associated with within-cohort changes in voiding diary parameters and a modest increase in maximum cystometric capacity. The retrospective, single-arm design precludes causal or treatment-specific inference, and patient-centered outcomes were not assessed. These findings are preliminary, exploratory, and hypothesis-generating rather than practice-informing.

## Introduction

Nocturnal enuresis is one of the most common lower urinary tract conditions in children and may negatively affect psychological well-being, self-esteem, family functioning, and quality of life (1). According to the International Children's Continence Society (ICCS), non-monosymptomatic nocturnal enuresis (NMNE) is defined as nocturnal enuresis accompanied by daytime lower urinary tract symptoms (LUTS), such as urgency, abnormal voiding frequency, daytime urinary incontinence, dysuria, or abnormal voiding patterns (2, 3). Compared with monosymptomatic nocturnal enuresis, NMNE is more frequently associated with underlying bladder dysfunction and usually requires targeted evaluation and management of daytime LUTS (1, 2).

Children with NMNE may present with reduced functional bladder capacity, detrusor overactivity, impaired bladder compliance, or incomplete bladder emptying. These abnormalities may contribute to both daytime symptoms and nocturnal urine leakage. Contemporary recommendations also emphasize the assessment of relevant comorbidities, including constipation, urinary tract infection, daytime incontinence, and sleep-disordered breathing, because these factors may influence treatment response (1, 4). Therefore, therapeutic strategies that improve bladder storage function may be clinically relevant in selected children with NMNE.

Mirabegron is a selective β3-adrenoceptor agonist that promotes detrusor relaxation during the storage phase and may increase bladder capacity without directly inhibiting voiding contraction. Pediatric pharmacokinetic and tolerability studies have provided a basis for weight-adjusted dosing (5), and mirabegron has been approved in the United States for pediatric neurogenic detrusor overactivity in children aged 3 years and older (6, 7). In addition, recent pediatric studies and systematic reviews have suggested that mirabegron may be a useful alternative or adjunctive therapy for children with refractory overactive bladder or neurogenic lower urinary tract dysfunction (8–15). However, direct evidence regarding its use in children with NMNE remains limited.

This retrospective real-world study aimed to describe exploratory within-cohort changes in clinical symptoms and urodynamic parameters after mirabegron-containing multimodal treatment in children with NMNE and reduced functional bladder capacity.

## Materials and methods

### Study design and reporting standard

This was a retrospective, single-center, observational study. The study was reported in accordance with the Strengthening the Reporting of Observational Studies in Epidemiology (STROBE) statement where applicable. Medical records of children with NMNE who received mirabegron at the outpatient clinic of West China Hospital of Sichuan University between July 2024 and March 2025 were reviewed.

### Patients

A total of 31 children were included. The mean age was 9.2 ± 2.5 years, with an age range of 5–14 years. The cohort included 13 boys (42.0%) and 18 girls (58.0%).

### Ethical considerations

This study was approved by the Ethics Committee of West China Hospital of Sichuan University (Approval No. 2025-6-3/No.1055). Written informed consent was obtained from the parents or legal guardians of all participants. The study was conducted in accordance with the Declaration of Helsinki.

### Inclusion and exclusion criteria

Patients were eligible for inclusion if they met all of the following criteria: (1) age ≥5 years; (2) nocturnal enuresis occurring at least twice per week; (3) symptom duration longer than 3 months; and (4) presence of daytime LUTS, including urinary urgency, increased voiding frequency, or daytime urinary incontinence.

Patients were excluded if they had any of the following: urinary tract anatomical abnormalities, active urinary tract infection at screening, monosymptomatic nocturnal enuresis, confirmed neurogenic bladder or neurological disorders affecting bladder function, previous urinary tract surgery, intellectual disability, clinically diagnosed psychiatric disorders that could affect symptom reporting or treatment adherence, incomplete medical records, treatment discontinuation, or loss to follow-up.

### Treatment protocol

Mirabegron was used as an add-on therapy to first-line treatment in children whose voiding diary suggested reduced functional bladder capacity. Estimated bladder capacity was calculated using the formula: estimated bladder capacity (mL) = 30 + age (years) × 30. Mirabegron was considered when the recorded maximum voided volume was lower than the estimated bladder capacity.

All children received oral mirabegron once nightly for 12 weeks. The dose was selected according to age and body weight. Children younger than 8 years with a body weight ≤24 kg received 25 mg once nightly, whereas children aged 8 years or older or weighing >25 kg received 50 mg once nightly. Dose adjustment was not included in the study protocol, and no patient underwent dose escalation or dose reduction during the treatment course.

Standard first-line management, including urotherapy and/or desmopressin when clinically indicated, was continued according to routine clinical practice. Because mirabegron was added to first-line treatment rather than used as monotherapy in all patients, no washout period was implemented. Treatment adherence was assessed based on outpatient records and parental reports during follow-up.

### Outcome measures

#### Voiding diary parameters

Voiding diary parameters were recorded before and after treatment. The following variables were analyzed: daily voiding frequency, nocturnal enuresis episodes, daytime incontinence episodes, and maximum voided volume.

#### Urodynamic parameters

Baseline urodynamic data were reviewed for all patients. Paired post-treatment urodynamic reassessment was performed when clinically indicated during follow-up. Maximum cystometric capacity was available before and after treatment in all 31 patients, whereas the availability of other paired urodynamic parameters varied.

#### Safety assessment

Adverse events assessed retrospectively from medical records and follow-up documentation. Records were reviewed for symptoms potentially associated with mirabegron, including headache, abdominal discomfort, constipation, palpitations, elevated blood pressure, urinary retention, and other newly documented symptoms.

### Diary-based enuresis episode reduction categories

Changes in nocturnal enuresis episodes were summarized using predefined diary-based reduction categories. Complete resolution was defined as no recorded nocturnal enuresis episodes after treatment. A reduction of at least 50% was categorized as meeting the predefined diary-based improvement threshold. A reduction of less than 50% was categorized as not meeting this threshold. These categories were used only to describe within-cohort diary changes and were not intended to represent a pharmacologic treatment response in the absence of a control group.

### Statistical analysis

Statistical analyses were performed using SPSS software, version 26.0. Continuous variables are presented as means ± standard deviations or medians with interquartile ranges, as appropriate. Normality of paired differences was assessed using the Shapiro–Wilk test. Normally distributed paired variables were compared using paired *t* tests, whereas non-normally distributed paired variables were compared using Wilcoxon signed-rank tests. Categorical variables are presented as frequencies and percentages. Mean differences and 95% confidence intervals were reported for paired comparisons. All tests were two-sided, and *P* < 0.05 was considered statistically significant.

## Results

### Patient characteristics and treatment regimens

Thirty-one children with non-monosymptomatic nocturnal enuresis were included in this study. The mean age was 9.2 ± 2.5 years, with an age range of 5–14 years. Thirteen patients were male and 18 were female. All patients received mirabegron-containing multimodal treatment for 12 weeks. Thirteen children received 25 mg once nightly, and 18 children received 50 mg once nightly. No dose escalation or dose reduction was performed during the treatment course. Detrusor overactivity was documented in 15 patients at baseline. Post-void residual urine volume was available in 9 patients and ranged from 7 to 100 mL. No treatment-related adverse events were documented in the available records (Table 1).

**Table 1:** Baseline characteristics and treatment regimens of the study population.

Characteristic	Value
Number of patients	31
Age, years	9.2 ± 2.5
Age range, years	5–14
Male sex	13 (42.0%)
Female sex	18 (58.0%)
Treatment duration	12 weeks
Mirabegron dose, 25 mg	13 (41.9%)
Mirabegron dose, 50 mg	18 (58.1%)
Dose escalation or reduction	0 (0%)
Detrusor overactivity at baseline	15 (48.4%)
PVR data available	9 (29.0%)
PVR among available records, mL	median 15, range 7–100
Documented treatment-related adverse events	0 (0%)

Data are presented as mean ± standard deviation, range, or *n* (%), as appropriate. PVR, post-void residual urine volume.

### Changes in voiding diary parameters

Statistically significant within-cohort changes were observed in voiding diary parameters after 12 weeks of mirabegron-containing multimodal treatment. Voiding frequency decreased from 6.90 ± 1.76 to 5.87 ± 0.99 episodes/day. Maximum voided volume increased from 282.90 ± 91.29 to 302.90 ± 76.78 mL. Daytime incontinence episodes decreased from 1.03 ± 1.05 to 0.29 ± 0.46 episodes/day. Nocturnal enuresis episodes decreased from 3.84 ± 1.27 to 1.35 ± 0.84 episodes/week. Because the study was uncontrolled, these changes should not be interpreted as treatment effects attributable to mirabegron. The results are summarized in Table 2.

**Table 2:** Changes in voiding diary parameters before and after 12 weeks of mirabegron-containing multimodal treatment.

Parameter	*n*	Before treatment	After treatment	Mean difference	*P* value
Voiding frequency, episodes/day	31	6.90 ± 1.76	5.87 ± 0.99	−1.03	<0.001
Maximum voided volume, mL	31	282.90 ± 91.29	302.90 ± 76.78	+20.00	<0.001
Daytime incontinence episodes/day	31	1.03 ± 1.05	0.29 ± 0.46	−0.74	<0.001
Nocturnal enuresis episodes/week	31	3.84 ± 1.27	1.35 ± 0.84	−2.48	<0.001

Data are presented as means ± standard deviations. Mean differences were calculated from paired individual-level data as post-treatment minus pre-treatment values. *P* values were obtained from paired tests selected according to the distribution of within-patient differences.

### Diary-based enuresis episode reduction categories

Based on the predefined diary-based categories, complete resolution of nocturnal enuresis episodes was observed in 5 patients (16.1%). A reduction of at least 50% was observed in 23 patients (74.2%), whereas 3 patients (9.7%) had a reduction of less than 50%. Overall, 28 of 31 patients (90.3%) met the predefined diary-based improvement threshold. Because all patients received mirabegron-containing multimodal treatment with first-line management in an uncontrolled setting, this proportion should not be interpreted as a pharmacologic response attributable to mirabegron. The results are summarized in Table 3.

**Table 3:** Diary-based categories of nocturnal enuresis episode reduction.

Diary-based category	Definition	*n* (%)
Complete resolution	No recorded nocturnal enuresis episodes after treatment	5 (16.1%)
≥50% reduction	Reduction of at least 50% in nocturnal enuresis episodes	23 (74.2%)
<50% reduction	Reduction of less than 50% in nocturnal enuresis episodes	3 (9.7%)
Meeting predefined diary-based improvement threshold	Complete resolution or ≥50% reduction	28 (90.3%)

### Changes in urodynamic parameters

Paired maximum cystometric capacity (MCC) data were available for all 31 patients. MCC increased from 271.94 ± 84.67 mL before treatment to 303.71 ± 85.93 mL after treatment, with a mean difference of +31.77 mL. The absolute increase was modest, and its direct clinical relevance could not be determined from the available data.

**Figure 1:**
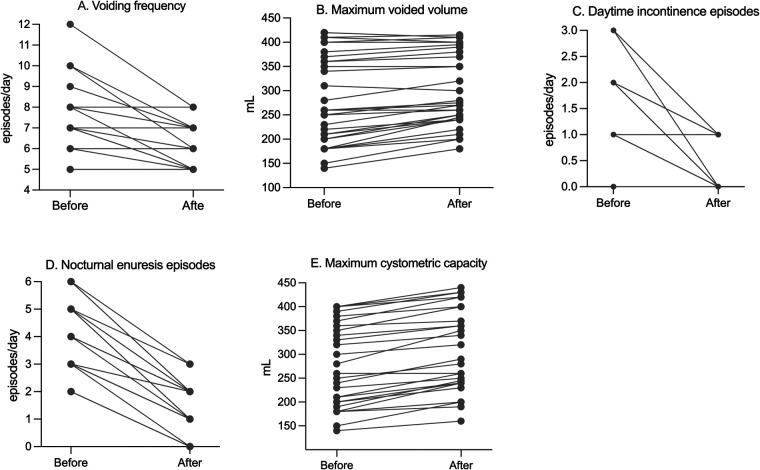
Individual pre- and post-treatment changes in voiding diary parameters and maximum cystometric capacity after 12 weeks of mirabegron-containing multimodal treatment. Paired individual-level plots show changes before and after treatment. Each gray line represents one patient, and the black line represents the group mean. Panels show changes in **(A)** voiding frequency, **(B)** maximum voided volume, **(C)** daytime incontinence episodes, **(D)** nocturnal enuresis episodes, and **(E)** maximum cystometric capacity. *P* values were obtained from paired tests selected according to the distribution of within-patient differences.

Repeat measurements of detrusor pressure at the end of filling (Pdet) and bladder compliance were available for four patients who underwent urodynamic reassessment during follow-up (Table 4 and Figure 1). Among these patients, Pdet decreased from 47.75 ± 7.37 to 30.00 ± 8.83 cmH_2_O, and bladder compliance increased from 29.25 ± 3.77 to 36.75 ± 2.75 mL/cmH_2_O. Given the extremely small sample, these data are presented strictly as descriptive within-subject observations and should not be considered evidence of urodynamic efficacy.

**Table 4:** Changes in urodynamic parameters before and after mirabegron-containing multimodal treatment.

Urodynamic parameter	*n*	Before treatment	After treatment	Mean difference	Interpretation
Maximum cystometric capacity, mL	31	271.94 ± 84.67	303.71 ± 85.93	+31.77	Within-cohort paired comparison
Detrusor pressure at the end of filling, cmH_2_O	4	47.75 ± 7.37	30.00 ± 8.83	−17.75	Descriptive only
Bladder compliance, mL/cmH_2_O	4	29.25 ± 3.77	36.75 ± 2.75	+7.50	Descriptive only

Data are presented as means ± standard deviations. Mean differences were calculated from paired individual-level data as post-treatment minus the pre-treatment values. Inferential statistics were applied only to MCC, for which paired data were available for 31 patients. Pdet and bladder compliance were available for only four patients and are presented strictly as descriptive within-subject observations.

### Safety

No treatment-related adverse events were documented in the available medical records or follow-up assessments. No patient discontinued treatment because of intolerance or adverse reactions. Available records did not document urinary retention or voiding difficulty leading to treatment discontinuation. However, post-void residual urine volume was not systematically recorded in all patients. Because adverse events were identified retrospectively from routine documentation, the absence of documented events should not be interpreted as evidence of safety.

## Discussion

In this retrospective real-world study, mirabegron-containing multimodal treatment was associated with within-cohort changes in voiding diary parameters and maximum cystometric capacity in children with NMNE. Because mirabegron was used as an add-on therapy to first-line management rather than as monotherapy, and because there was no control group, the observed changes should be interpreted strictly as exploratory, hypothesis-generating observations within a multimodal and uncontrolled treatment setting. Repeat measurements of Pdet and bladder compliance were available only in a small subset of patients; therefore, these urodynamic findings should be considered descriptive rather than confirmatory.

NMNE differs from monosymptomatic nocturnal enuresis because it is accompanied by daytime LUTS and is frequently associated with bladder dysfunction. ICCS recommendations emphasize that daytime LUTS and relevant comorbidities should be addressed before or alongside nocturnal enuresis-directed treatment (1, 2). In children with NMNE, reduced functional bladder capacity, detrusor overactivity, impaired bladder compliance, and incomplete bladder emptying may contribute to persistent nocturnal and daytime urinary symptoms. Therefore, management of the underlying lower urinary tract dysfunction is an important component of NMNE treatment.

In the present study, mirabegron was added to first-line management in children whose voiding diaries suggested reduced functional bladder capacity. The observed changes cannot be attributed solely to mirabegron because standard urotherapy, desmopressin when clinically indicated, behavioral modification, placebo response, regression to the mean, spontaneous improvement, and natural maturation of bladder function may also have contributed. Therefore, the proportion meeting the diary-based improvement threshold reported in this study should not be interpreted as a pharmacologic response attributable to mirabegron.

Mirabegron activates β3-adrenoceptors in the detrusor muscle, leading to bladder relaxation during the storage phase. Pediatric pharmacokinetic studies have supported the feasibility of pediatric dosing strategies (5), and a phase 3 dose-titration study evaluated mirabegron in children and adolescents with neurogenic detrusor overactivity (8). Mirabegron has also been studied in pediatric overactive bladder and neurogenic detrusor overactivity. Therefore, the present study does not establish a new therapeutic paradigm but provides preliminary observational data in a specific subgroup of children with NMNE. The findings should be considered exploratory and hypothesis-generating.

The clinical outcomes in this study were primarily based on voiding diary records. Although voiding diaries are commonly used in the evaluation of pediatric lower urinary tract symptoms, they are subject to recall bias, parental reporting variability, incomplete recording, and day-to-day symptom fluctuation. Therefore, the observed changes in diary-based parameters should be interpreted as approximate within-cohort observations rather than precise estimates of treatment effect.

The increase in MCC was observed in the full cohort and may reflect a change in bladder storage capacity. This direction is consistent with previous pediatric reports describing bladder capacity or storage parameters after mirabegron treatment in children with refractory overactive bladder or neurogenic lower urinary tract dysfunction (9–14). A recent systematic review of mirabegron for pediatric refractory idiopathic overactive bladder also reported symptom improvement and favorable safety findings, while emphasizing that the evidence remains limited and largely uncontrolled (15). In the present study, however, the absolute increase in MCC was modest. Without validated symptom scores, sleep quality measures, psychosocial assessments, family impact assessments, or quality-of-life instruments, it remains uncertain whether this urodynamic change corresponded to a clinically meaningful benefit for patients or families.

Changes in Pdet and bladder compliance were observed only in four patients with available repeat urodynamic reassessment. In this small subset, the observed decrease in Pdet and increase in bladder compliance may be compatible with altered bladder storage dynamics, but these findings should be interpreted strictly as descriptive within-subject observations. They should not be regarded as robust evidence of urodynamic efficacy and require confirmation in larger cohorts with standardized repeat urodynamic assessment.

No treatment-related adverse events were documented; however, this finding should be interpreted cautiously because adverse events were assessed retrospectively and the sample size was small. The absence of documented adverse events should not be interpreted as evidence of safety. Mild events, including blood pressure changes, headache, constipation, palpitations, or transient voiding symptoms, may have been underreported in routine clinical documentation. The safety of mirabegron-containing treatment in this population should therefore be evaluated prospectively using standardized monitoring of blood pressure, heart rate, post-void residual urine volume, and patient-reported adverse symptoms.

This study has several limitations. First, it was a retrospective single-arm study without a placebo or active control group; therefore, causal inference regarding the efficacy of mirabegron could not be established. Second, mirabegron was used as an add-on therapy to first-line management rather than as monotherapy in all patients, and the independent effect attributable to mirabegron could not be isolated. Third, standard urotherapy, concomitant desmopressin, behavioral modification, placebo response, regression to the mean, spontaneous improvement, and natural maturation of bladder function may have contributed to the observed changes. Fourth, although maximum cystometric capacity was available before and after treatment in all patients, repeat measurements of Pdet and bladder compliance were available only in a small subset of patients; therefore, conclusions regarding storage pressure and bladder compliance should be interpreted cautiously. Fifth, post-void residual urine volume, uroflowmetry parameters, constipation status, sleep disorders, prior treatment history, and quality-of-life outcomes were not systematically assessed in all patients, introducing residual confounding and limiting interpretation of response variability across the cohort. Sixth, the study did not include validated patient-centered outcome instruments, including symptom burden scores, sleep quality measures, psychosocial functioning, family impact, or quality-of-life measures; therefore, the clinical meaningfulness of the observed urodynamic changes remains uncertain. Finally, adverse events were assessed mainly through medical records and follow-up, which may have underestimated mild, transient, or infrequent adverse events.

Despite these limitations, the present study provides preliminary real-world observational data in a clinically relevant subgroup of children with NMNE. The findings describe within-cohort changes in diary-based urinary symptoms and bladder storage capacity within a multimodal treatment setting, but they should be interpreted as exploratory and hypothesis-generating rather than clinically actionable or practice-informing. Confirmation requires prospective controlled studies with standardized safety monitoring, systematic baseline phenotyping, and validated patient-centered outcome measures.

## Conclusion

In this retrospective, single-arm, uncontrolled cohort, mirabegron-containing multimodal treatment was associated with within-cohort changes in voiding diary parameters. Maximum cystometric capacity also showed a modest increase. These findings do not establish the efficacy or safety of mirabegron and should not be considered practice-informing. Descriptive changes in Pdet and bladder compliance were observed in only four patients and should be interpreted with particular caution. The findings are preliminary, exploratory, and hypothesis-generating and require confirmation in prospective controlled studies with standardized safety monitoring, systematic baseline phenotyping, and validated patient-centered outcome measures.

## Data Availability

The original contributions presented in the study are included in the article/Supplementary Material, further inquiries can be directed to the corresponding authors.
